# The immune cell dynamics in the peripheral blood of cHL patients receiving anti-PD1 treatment

**DOI:** 10.3389/fonc.2025.1518107

**Published:** 2025-03-13

**Authors:** Vanessa Cristaldi, Lodovico Terzi di Bergamo, Lucrezia Patruno, Marinos Kallikourdis, Giada Andrea Cassanmagnago, Francesco Corrado, Eleonora Calabretta, Adalgisa Condoluci, Martina di Trani, Daoud Rahal, Gianluca Basso, Clelia Peano, Alex Graudenzi, Marco Antoniotti, Davide Rossi, Carmelo Carlo-Stella

**Affiliations:** ^1^ Department of Biomedical Sciences, Humanitas University, Milano, Italy; ^2^ Laboratory of Experimental Hematology, Institute of Oncology Research, Bellinzona, Switzerland; ^3^ Department of Health Science and Technology, Swiss Federal Institute of Technology (ETH Zürich), Zurich, Switzerland; ^4^ Department of Informatics, Systems and Communication of the University of Milan-Bicocca, Milan, Italy; ^5^ Adaptive Immunity Lab, IRCCS Humanitas Research Hospital, Milan, Italy; ^6^ Department of Oncology and Hematology, Humanitas Cancer Center, IRCCS Humanitas Research Hospital, Milan, Italy; ^7^ Oncology Institute of Southern Switzerland, Ente Ospedaliero Cantonale, Bellinzona, Switzerland; ^8^ Department of Pathology, IRCCS Humanitas Research Hospital, Milan, Italy; ^9^ Institute of Genetics and Biomedical Research, UoS of Milan, National Research Council, Milan, Italy; ^10^ Institute of Molecular Bioimaging and Physiology, Consiglio Nazionale delle Ricerche (IBFM-CNR), Milan, Italy; ^11^ Bicocca Bioinformatics, Biostatistic, Bioimaging Centre (B4), Università degli Studi di Milano-Bicocca, Milan, Italy

**Keywords:** Hodgkin (cHL), single-cell analysis, TCR - T cell receptor, immunotherapy, refractoriness

## Abstract

Checkpoint blockade therapy (CBT) involving anti-PD1 antibodies represents the standard approach for cHL patients who do not respond to second-line therapy. Nonetheless, only 20% of relapsed/refractory (R/R) cHL patients treated with CBT achieve complete remission. In this study, we extensively examined the immune dynamics in eight R/R cHL patients treated with CBT, consisting of four complete responders (CR) and four experiencing disease progression (PD), by single cell analysis of peripheral blood mononuclear cells (PBMCs). Our unique approach encompassed longitudinal analysis with three time points, providing a comprehensive understanding of the evolving immune responses during anti-PD1 therapy. Through gene expression profiling, we identified a stable and distinctive KLRG1+/FOS+/JUN+/GZMA+/CD8+ T cell phenotype in patients achieving complete responses. This specific CD8+ T cell subset exhibited sustained activation, underscoring its potential pivotal role in mounting an effective immune response against cHL. Furthermore, T cell receptor (TCR) analysis revealed that in responder patients there is clonal expansion between TCR clonotypes specifically in the KLRG1+/FOS+/JUN+/GZMA+/CD8+ T cell subset. Our longitudinal study offers unique insights into the complex immune dynamics of multiply relapsed/highly pre-treated cHL patients undergoing anti-PD1 therapy.

## Introduction

Classic Hodgkin Lymphoma (cHL) is a distinct subtype of lymphoma characterized by the presence of Hodgkin and Reed-Sternberg (HRS) cells within a background of inflammatory infiltrate (1, 2). The immune microenvironment plays a critical role in the pathogenesis and progression of cHL (3, 4). HRS cells evade immune surveillance through various mechanisms, including the expression of programmed death-ligand 1 (PD-L1) and secretion of immunosuppressive cytokines (5).

The tumor microenvironment of cHL is characterized by a diverse array of immune cells, including T cells, B cells, macrophages, eosinophils, and mast cells (6). CD4+ T helper cells are abundant within cHL tumors (7, 8), but they often exhibit an exhausted phenotype marked by high expression of immune checkpoint receptors, such as programmed cell death protein 1 (PD-1) (9). Regulatory T cells (Tregs) are also enriched within cHL lesions, contributing to immunosuppression and tumor immune evasion (10, 11).

Immunotherapy has revolutionized the treatment landscape of cHL, particularly through the use of immune checkpoint inhibitors targeting the PD-1/PD-L1 axis (12, 13). Pembrolizumab and nivolumab, anti-PD-1 antibodies, have demonstrated remarkable efficacy in relapsed or refractory cHL, leading to durable responses in a significant proportion of patients (14, 15).

The rationale for targeting PD-1/PD-L1 signaling in cHL stems from the overexpression of PD-L1 on HRS cells and the presence of PD-1-expressing exhausted T cells within the tumor microenvironment. By blocking the PD-1/PD-L1 interaction, immune checkpoint inhibitors unleash the cytotoxic activity of T cells against cHL cells, leading to tumor regression (16).

More recently, clinical trials have demonstrated the efficacy of immune checkpoint inhibitors as monotherapy or in combination with chemotherapy in additional settings (17), including first relapsed/refractory disease (18), frontline treatment and as maintenance therapy following transplantation (19, 20). However, not all patients respond to immunotherapy, highlighting the need for accessible biomarkers to identify responders and strategies to overcome resistance mechanisms.

Our research aims to investigate whether alterations in the peripheral blood T cell pool can serve as a biomarker for predicting response to immunotherapy in patients with R/R cHL. Through single-cell RNA sequencing (scRNA-seq) (21) and single cell TCR sequencing (scTCR-seq) (22), we obtained high-resolution transcriptomic profiles of peripheral blood mononuclear cells of patients with R/R cHL, deconvoluted the T cell subtypes and clonotypes and correlated them with response to immunotherapy.

## Materials and methods

### Patients recruitment

The study was retrospective in nature and included a total of eight patients with R/R cHL, aged >18 years, who were enrolled in the CheckMate 205 trial (23) and treated with Nivolumab (**Table 1**). Staging and disease response were assessed according to the Lugano 2014 criteria (24). The study was conducted in accordance with International Conference on Harmonization for Good Clinical Practice guidelines and the Declaration of Helsinki (25). Written informed consent was obtained from all patients before enrollment. Patients were administered Nivolumab at a dose of 3 mg per kilogram of body weight (26) every 2 weeks at Humanitas Research Hospital.

**Table 1:** Patient feature overview table.

Overall	
n	8
Patients characteristics	
Age (median[IQR])	33.17 [18.99-51.05]
Sex	7 (87.5)
Extranodal (%)	5 (62.5)
Bulky (%)	0 (-)
B Symptoms (%)	2 (25%)
Previous Treatments	
Number of prior treatments (median [IQR])	4.75 [3.00-7.00]
Autologous stem cell transplantation (%)	6 (75.0)
Radiotherapy (%)	2 (25.0)
Brentuximab Vedotin (%)	8 (100.0)
BV and auto-SCT (%)	6 (75.0)
Advanced stage (%)	7 (87.5)
Refractory to the last treatment (%)	4 (50.0)
Anti-PD1 Treatment	
Duration (median[IQR])	16.17 [5.63-26.83]
Number of cycles (median[IQR])	31.87 [9-55]

**Figure 1:**
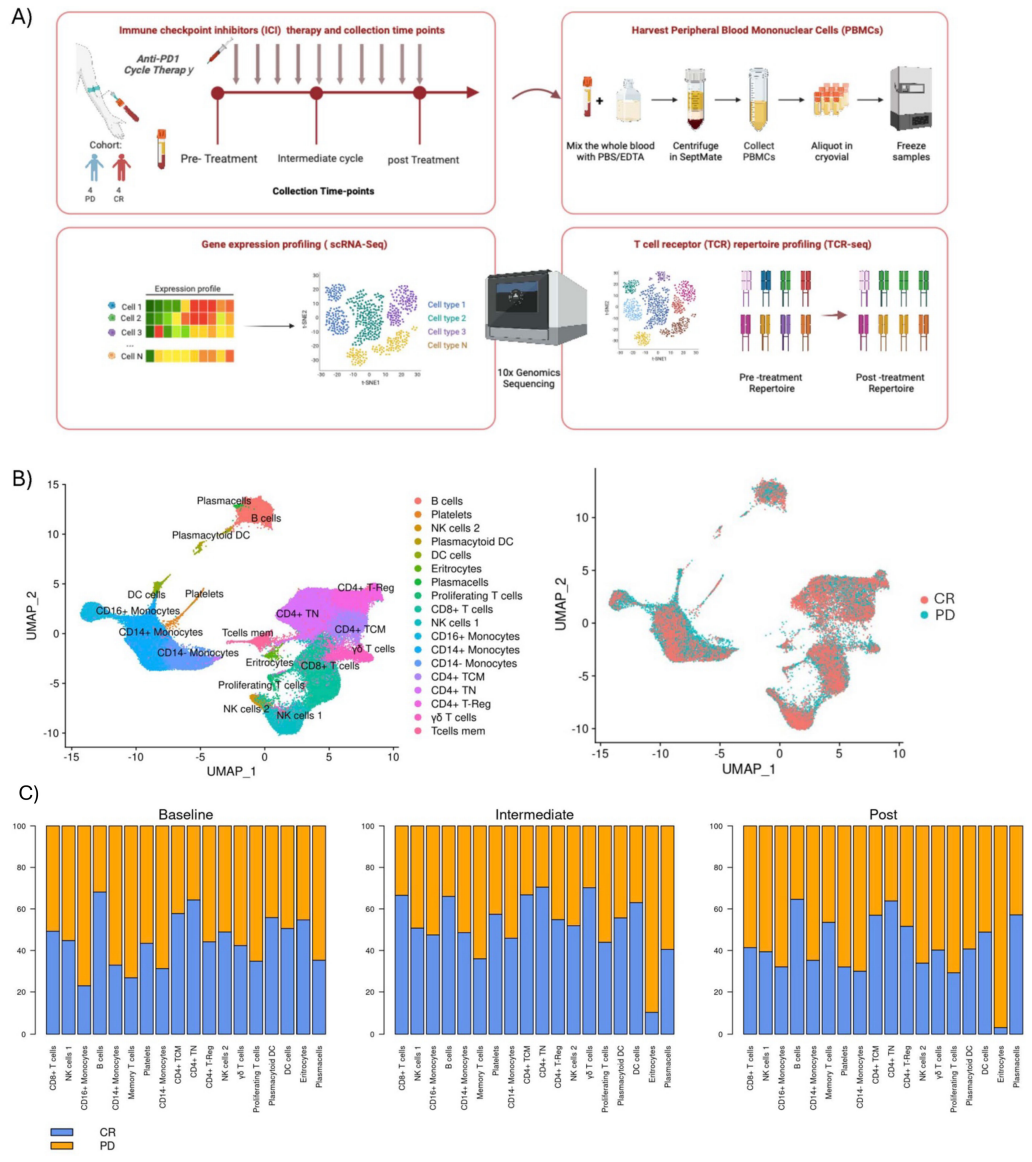
Overall study design and clustering: **(A)** Eight patients were included in the study, from whom peripheral blood were collected at three different time points: before treatment initiation (Pre-treatment), after the 4th cycle of therapy (Post 4 cycles), and following the 8th or 9th cycles of treatment (Post 8-9 cycles). Four exhibited progression disease (PD), and four achieved a complete response (CR). PBMCs were assayed by single-cell RNA sequencing (scRNA-seq) and T-cell receptor sequencing (TCR-Seq). **(B)** Two-dimensional similarity map (UMAP projection) of single-cell gene expression of all cells from 8 r/r cHL. Cells are colored according to the PhenoGraph cluster (left) and by class of final response to treatment (right) **(C)** Barplots with proportion of each cell cluster, according to time-point for both the PD and CR class.

For each patient, we collected three samples of PBMCs: before cycle 1 (Pre-Treatment stage), before cycle 5 (Intermediate stage) and before cycle 10 (Post Treatment stage) with anti-PD1 immunotherapy (except for one PD patient whose cycle 5 we do not have) (**Figure 1**). These patients were categorized into two response groups based on their response at cycle 10: four patients who remained in progressive disease (PD) and four patients who achieved a complete response (CR).

### Library preparation and single-cell RNA sequencing

In total an average of 5000 cells per sample were loaded into a Chromium Single Cell 3’ Chip kit v2 (PN-120236) and processed according to the Chromium Single Cell 3’ Reagent kit v2 User Guide. Libraries were constructed using the Single Cell 3’ Library and Gel Bead Kit v2 (PN-120237) and Chromium i7 Multiplex Kit (PN-120262). All single cell libraries were pooled and sequenced on Illumina NextSeq550. CellRanger software (v2.1.0) was used to demultiplex the raw data, generate quality metrics, and generate per-gene count data for each cell. Count data from 8 r/r cHL samples were pooled together, including only features with expression data found in at least 3 cells, and with at least 200 features observed, cells were filtered out if they had >20% reads aligning to mitochondrial genes, or if they had >2500 features detected. Clustering was performed using the FindClusters function from the Seurat (27) package in R. The total list of genes used for clusters annotation was composed of previously described genes signatures (18) and a list of manually curated markers (see attached **Supplementary Data**). Differential expression between patients and between class of response were performed in selected cell populations.

### T cell receptor sequencing

The same samples were also analyzed for scTCR-sequencing. 10X Genomics standard protocol was applied and the reagents for the Chromium Single-cell 5’ Library and V(D)J library (v2.0 Chemistry) were used. Barcoded VDJ libraries were pooled and sequenced by an Illumina NextSeq 550 Sequencer. Single-cell TCR sequencing data were processed by the Cell Ranger software pipeline (v2.1.0; 10X Genomics).The TCR sequence data were processed using Scirpy (28) (Single-Cell Immune Receptor Profiling). Scirpy was also utilized to identify and quantify clonal diversity, ascertain the presence of dominant T cell clones, and assess repertoire stability over time.

### Statistical analysis

Statistical analyses were conducted using GraphPad Prism (version 9) in conjunction with R version 4.0.3. Specifically, for group comparisons, the t-test and Mann-Whitney test were employed, depending on the data distribution and specific analysis conditions.

## Results

### Patients’ characteristics and response to therapy

Patients (n=8) had a median age of 33 years (interquartile range 19-51), and 7 (87.5%) were males. Before treatment, 87.5% of patients (n=7) showed an advanced disease (Stage IIB-IV), with extranodal involvement present in 5 (62%) cases. Notably, the study group consisted of highly pre-treated patients, with a median of 5 previous lines of therapy (**Table 1**). Six (75%) patients had previously received autologous stem-cell transplantation and all had received Brentuximab-Vedotin. Patients were treated with Nivolumab for a median of 16 cycles (interquartile range 6-27) and classified as responsive or unresponsive to the PD1 blockade based on the best overall response (BOR) and the duration of response (DOR). The best overall response was defined as the best response between the first dose and progression (29). Patients who achieved a best complete response were considered responsive (n=4) while patients with primary progressive disease (n=1) or best partial response lasting less than 6 months (n=3) were considered unresponsive. Among patients achieving CR, 3 (75%) underwent consolidative allogeneic transplantation (allo-SCT). The remaining patient exhibited a prolonged DOR (15.5 months). Patients achieving a best partial response (n=3) showed a limited median DOR of 4 months. No gross difference in the distribution of main clinical features was observed between the two groups (**Supplementary Table 1**).

### Immune cells dynamics during nivolumab therapy

The size of our cohort, although limited, was deemed appropriate due to the specific clinical characteristics of the patients included in the study. Our cohort consisted exclusively of patients with Hodgkin lymphoma who were refractory to first-line treatments, representing a particularly rare and clinically significant subgroup of the disease. This targeted selection allowed us to obtain data representative of this highly relevant population, despite the small sample size. Importantly, our analysis included data collected from multiple time points for each patient, which increased the robustness of our observations and provided dynamic insights into the behavior of this population. We acknowledge the inherent limitations of the sample size and we consider this an observational study. Indeed, our work serves as an exploratory investigation that lays the groundwork for future studies in larger cohorts.

We performed an integrated analysis of all samples, comparing PB cell types pre-treatment, during treatment, and post-treatment between responders and non-responders (**Figure 1B**). Importantly, for each patient, data were collected at three distinct time points, enabling robust longitudinal profiling of the immune response. We observed a trend where lymphocyte populations, such as naïve CD4+ T cells (CD4+TN), CD4+ central memory (CD4+CM), CD8+ T cells, γδT cells, and B cells, were enriched in responding patients across all treatment phases, suggesting a coordinated response of these cells against tumor cells, though no statistical significance was achieved. Conversely, populations such as CD14+ and CD14-/CD16+ monocytes were more abundant in non-responders both during therapy and already at the pre-treatment stage (**Figure 1C**). These findings align with Cader et al. (37), that obtained a peripheral immune signature of PD-1 blockade responsiveness in 56 patients treated in the phase II CheckMate 205 clinical trial (NCT02181738). Their results demonstrated that PD-1 blockade was most effective in patients with a diverse baseline TCR repertoire, accompanied by an expansion of singleton clones during treatment. Importantly, they reported a significant increase in CD4+ TCR diversity, particularly in patients who achieved complete responses. This highlights the critical role of CD4+ T cells in the effectiveness of PD-1 blockade. In light of these findings, our study aims to shift the focus to the role of CD8+ T cells in the immune response to treatment. Understanding their dynamics, clonal expansion, and studying their cytotoxic potential to provide a comprehensive understanding of the immune landscape in classical Hodgkin lymphoma, especially in refractory cases.

Furthermore, following the application of signatures associated with the type I interferon response to inflammation on patients’ peripheral blood mononuclear cells (PBMCs), a noteworthy observation emerged. Specifically, in PD patients, the monocytic subgroup demonstrated a remarkably distinct module score linked to the pathway associated with response to type I interferon stimulation during the pre-treatment phase (GO: 0034340 from human GSEA dataset) (**Supplementary Figure 5**).

### Enhanced activation and effector gene expression in CD8+ T Cells of responding patients

**Figure 2:**
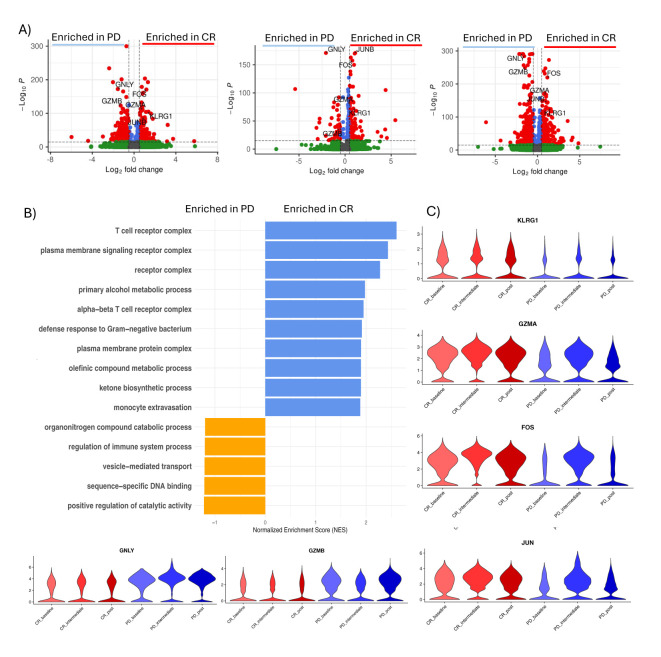
CD8+ T cells activation in CR patients. **(A)** Volcano plot showing differentially expressed genes between CD8+ T cells of responsive patients (CR) vs non responsive patients (PD) in pre-treatment (left), intermediate (middle) and post-treatment (right). Significant genes are labeled in red (P value <0.05 and absolute log2 fold change ≥0.5). Selected important genes for T cells are labelled. **(B)** Gene Set Enrichment Analysis (GSEA) of differentially expressed genes between CR and PD patients for each time-point. **(C)** Violin plot showing the expression of GZMA, FOS, KLRG1, JUN, GNLY and GZMB in each group of patients during time.

Focusing our attention on CD8+ T cells, we conducted differential expression gene (DEG) analysis across all peripheral blood mononuclear cell (PBMC) populations. (**Supplementary Figures 3A–C**). Over time, CD8+ T cells from responsive patients (CR) exhibited a significant (cut-off for log2FC >2; P=10e-32) upregulation of effector/activation genes such as *KLRG1*, *FOS*, *JUN*, and *GZMA* (**Figure 2**). Co-expression of *KLRG1*, *FOS*, *JUN*, and *GZMA* remained constant during all time-points in responsive patients. Using genes identified from differential gene expression analyzes between CD8+ cells from treatment-responsive and non-responsive patients, a Gene Set Enrichment Analysis (GSEA) was conducted to identify the gene pathways involved. Complete response (CR) patients showed significant enrichment (p value adjust < 0.05) of gene sets related to T cell receptor (TCR) activation, cellular activation and T cell activation, which was also confirmed after treatment. At midcycle, CR patients showed significant up-regulation of processes related to T cell activity, including the assembly and activity of Major Histocompatibility Complex (MHC) class I.

### TCR clonal expansion observed in KLRG1+ FOS+ JUN+ GZMA+ CD8+ T subclusters of responsive patient’s class

**Figure 3:**
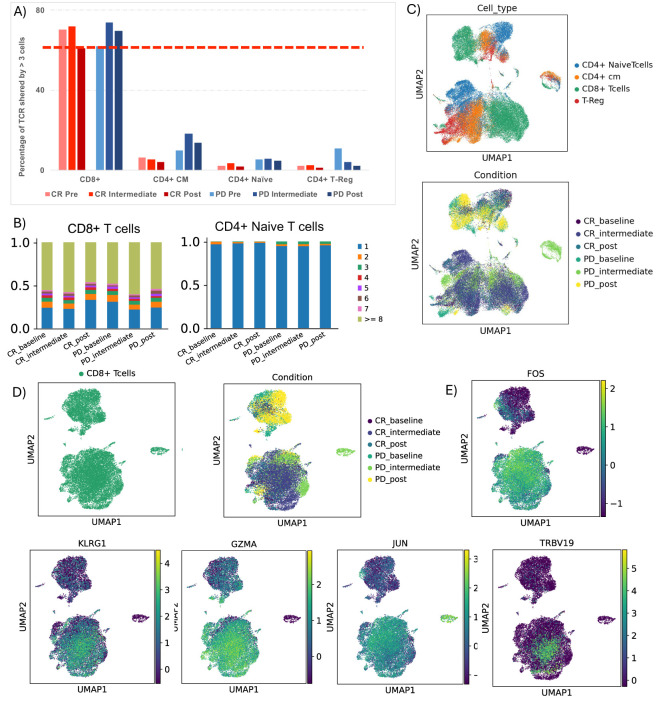
TCR clonotype tracking and clonotype expansion during time. **(A)** Barplot showing the percentage of T cell receptor (TCR) shared by more than 3 cells in each cluster, comparing CR and PD. **(B)** Detailed TCR clonotype expansion from 0 to 1.0 (100%) for CD4 central memory, CD8 T cells and Naïve T cells, in all patient conditions. **(C)** UMAP showing TCR clusters in all cluster based on their CD3 region sequence, colored by cell type, and by groups of patients during time. **(D)** UMAP showing TCR clusters in CD8 T cells cluster based on their CD3 region sequence, colored by cell type, and by groups of patients during time. **(E)** UMAP showing FOS, KLRG1, GZMA, JUN and TRBV19 genes in CD8+ T cells cluster.

We conducted immunophenotypic analysis of lymphocytes through T cell receptor repertoire sequencing (TCR-seq) using 10x Genomics to better understand the immune response in patients. Our analysis revealed a notable phenomenon of clonal expansion, where TCR structures were shared by more than three cells, predominantly present in the CD8+ T cell cluster across both response groups (**Figure 3A**). Interestingly, we observed that non-responsive patients exhibited greater clonal expansion in central memory (CM) CD4+ T cells, particularly in the intermediate stage. In contrast, naïve CD4+ T cells primarily showed individual clone expansions during treatment, without an overall increase in clones over time (**Figure 3**). Notably, our study highlights that even at baseline, responders displayed less clonal expansion in CD4+ T cell classes (CD4 CM, CD4 Naïve, and CD4 Treg) compared to non-responders, this highlight what showed by Cader et al. A more detailed analysis of TCR structures revealed an overlap of the expanded CD8+ TCR clonotypes in responsive patients with a CD8+ T cell subcluster characterized by the KLRG1+ and GZMA+ phenotype, previously identified in scRNA-seq analysis. This overlap suggests a correlation between the clonal expansion of CD8+ TCRs and the T cell phenotype associated with treatment response (**Figure 3**). To further evaluate this correlation, we applied a TCR activation signature (GOCC: “Alpha Beta T cell receptor complex” from the human GSEA dataset) to the dataset (**Supplementary Figure 4**). The results indicate that the module score is higher in CD8+ TCRs from responding patients, reinforcing the association between clonal expansion of CD8+ TCRs and treatment response in patients with complete response (CR).

## Discussion

In this genetic and immunophenotypic profiling study of PBMC populations we investigated the immune dynamics of patients with relapsed/refractory classical Hodgkin lymphoma (r/r cHL).

It is essential to recognize that these patients, despite having demonstrated a non-response to previous treatments, were still subjected to various therapies before anti-PD1 treatment (**Table 1**). Of relevance, our cohort included heavily pre-treated patients, who received a high number of therapies prior to PD-1 blockade. Given these considerations, our study observed the KLRG1+, GZMA+ phenotype in CD8+ T cells in patients who achieved a complete response (CR) to anti-PD1 therapy.

These findings suggest that this specific subset of CD8+ T cells could play a fundamental role in predisposing the patient to a better response to anti-PD1 treatment. Furthermore, it is interesting to note that this phenotype remained stable throughout time, suggesting consistent and robust activity of CD8+ T cells with this specific phenotype.

These genes are known to play a role in the activation of CD8+ T cells, enhancing their anti-tumor function (30). *KLRG1*, Killer Cell Lectin-Like Receptor G1, is predominantly expressed by CD8+ effector T cells and influences immunological memory (31, 32). The genes FOS and JUN indicate an activation and differentiation of CD8+ T cells toward a more effector state (33) and GZMA is involved in cytotoxic mechanisms (34).

The significant Increase in the expression of genes such as GZMB (granzyme B) and GNLY (granulysin) in CD8+ T cells of patients unresponsive to anti-PD1 treatment could reflect an attempt by the immune system to fight the tumor through the activation of CD8+ T cells. However, despite the increase in these genes, the lack of response may suggest that other factors, such as the immunosuppressive tumor microenvironment or the presence of immune evasion mechanisms, may limit the effectiveness of the cytotoxic activity of CD8+ T cells.

Furthermore, the expression of GZMB and GNLY could indicate a state of chronic activation of CD8+ T cells (35), which could lead to exhaustion and dysfunction of T cells, hindering their effective role in eliminating tumor cells (36). Therefore, these findings highlight the importance of a deeper understanding of the mechanisms involved in Hodgkin lymphoma and the response to immunotherapies to develop more effective strategies for the treatment of non-responsive patients.

To test the exhaustion status of CD8+ T cells, we analyzed the expression of transcription factors TCF-1 (TCF7) and TOX, which are critical markers for assessing the terminal exhaustion of T cells. Our data show that TCF-1 is upregulated, indicating the presence of progenitor exhausted T cells, while TOX expression is not significantly elevated. These findings suggest that the CD8+ T cells in our cohort are not terminally exhausted. We have included these results in **Supplementary Figure 6** to clarify the exhaustion status and its potential implications for anti-PD-1 treatment failure. Regarding memory phenotypes present in CD8+ T cell cluster, we used the module score to assess whether CD8+ T cells in patients with complete response (CR) and progressive disease (PD) predominantly exhibit effector memory (EM) or CD45RA-reexpressing effector memory (EMRA) phenotypes. The analysis, presented in **Supplementary Figure 7**, shows no significant difference between the two patient groups. This lack of distinction could be attributed to the refractory nature of our cohort, with patients undergoing multiple lines of treatment, potentially masking classic phenotype distinctions.

Our results are in contrast with the study by Reinke et al. (40)which instead reported a decrease in peripheral CD8+ T cells during anti-PD-1 therapy. Our results reveal distinct dynamics in circulating CD8+ T cells, particularly when stratifying patients based on therapeutic response. This dynamic suggests that effective PD-1 blockade triggers a systemic immune response in responders, which peaks during treatment and subsides post-treatment as tumor burden decreases. In contrast, the higher percentage of CD8+ T cells observed in PD patients post-treatment does not indicate successful immune activation. Instead, this trend may result from the persistence of less functional or exhausted CD8+ T cells in circulation, reflecting the inability of the immune system to effectively target and clear tumor cells. This interpretation is consistent with the lack of a significant CD8+ T-cell expansion during intermediate cycles in PD patients, highlighting a fundamental difference in the immune dynamics of responders versus non-responders.

About the TCR analysis, it is interesting to note that we did not observe significant differences in clonal expansion between CD8+ T cells from CR and PD patients. However, the most significant aspect of this TCR data lies in the precise overlap between TCR clonotypes exhibiting clonal expansion (>3 cells) in CR patients and the KLRG1+, FOS+, JUN+, GZMA+ CD8+ T cell subset. This detail is crucial as it suggests that this specific subset of CD8+ T cells could be relevant in mediating the immune response in patients who respond to anti-PD1.

This alignment strengthens the robustness of our findings by demonstrating a consistent relationship between TCR specificity and cellular characteristics. Further research is needed to fully elucidate the functional significance of these TCR phenotypic associations and to determine how they can be exploited for therapeutic benefit.

Our findings are also consistent with the recent work of Chen et al. (38), who provide a comprehensive characterization of cHL patients using scRNA-seq, TCR sequencing, and validated functional studies by flow cytometry. Although Chen et al. analyzed lymph node biopsies, focusing on the tumor microenvironment, while our study investigates PBMCs, both studies report expanded CD8+ T cell clonotypes with a nuanced exhaustion profile. In line with our findings, Chen et al. observe a lack of terminal exhaustion markers, such as TOX, but report the expression of genes such as EOMES, TIGIT, and HAVCR2, indicative of partial exhaustion or adaptation. These parallels, despite differences in tissue origin and cohort characteristics, underscore the complexity of the immune landscapes of cHL and the need to consider both local and systemic immune compartments for a comprehensive understanding of the pathophysiology of the disease.

Our findings complement those of Michot et al. (39) who used multiplex immunohistochemistry to analyze immune dynamics at the tumor site before and after anti-PD-1 therapy. While Michot et al. observed a decrease in CD8+ T cells and LAG-3 overexpression in the tumor microenvironment, our study focuses on PBMCs, providing insights into systemic immune responses. Notably, in complete responders (CR), we detected a transient increase in circulating CD8+ T cells during treatment, likely reflecting systemic immune activation. Conversely, in progressive disease (PD) patients, CD8+ T cells decreased during treatment cycles, followed by a late-stage increase, possibly due to compensatory mechanisms. These differences highlight the distinct immune dynamics between tumor-localized and systemic compartments, underscoring the importance of analyzing both to fully understand the immune response in cHL.

Overall, this work provides further evidence of the dynamic and complex nature of the immune response induced by anti-PD1 therapy. The expansion of specific CD8+ T cell clones, especially within responding patients, suggests their potential role in driving an effective anti-tumor response. However, the simultaneous clonal expansion of additional CD8+ T cell clones in all patients indicates a more complex interaction of immune cell populations during treatment.

Further investigation into the functional properties of these expanded T cell clones and their interactions with other immune cells would be essential to fully understand their contribution to treatment outcomes and guide the development of personalized immunotherapeutic strategies for cancer patients undergoing anti- PD1 therapy.

## Data Availability

The sequencing data generated in this study have been deposited in the Gene Expression Omnibus (GEO) under the accession number GSE290026. The data are publicly available and can be accessed at https://www.ncbi.nlm.nih.gov/geo/.
