# Supplementary material for: The immune cell dynamics in the peripheral blood of cHL patients receiving anti-PD1 treatment
Source: Front Oncol. 2025 Mar 13;15:1518107. doi: 10.3389/fonc.2025.1518107 (PMC11966435; doi:10.3389/fonc.2025.1518107)
Supplement: Supplementary file 1 [file DataSheet1.pdf]

	Responders	Non-Responders	p*
**n**	4	4	
**Age (median [IQR])**	37.11 [32.00, 41.76]	28.63 [23.50, 34.83]	0.386
**Male (%)**	4 (100.0)	3 (75.0)	1.000
**Prior Therapies (median [IQR])**	4.50 [3.75, 5.50]	5.00 [4.75, 5.00]	0.757
**AutoSCT(%)**	4 (100.0)	2 (50.0)	0.414
**Radiotherapy (%)**	1 (25.0)	1 (25.0)	1.000
**BV (%)**	4 (100.0)	4 (100.0)	NA
**BV+auto (%)**	4 (100.0)	2 (50.0)	0.414
**Advanced stage (%)**	3 (75.0)	4 (100.0)	1.000
**Refractory to first line (%)**	2 (50.0)	3 (75.0)	1.000
**Refractory to last line (%)**	2 (50.0)	2 (50.0)	1.000
**Extranoddal (%)**	2 (50.0)	3 (75.0)	1.000
**B-Symptoms (%)**	1 (25.0)	1 (25.0)	1.000

Supplementary table 1
Table of main clinical covariates between the two groups of patients. p*= Categorical variables are assessed using the Fisher's test (p-value), while continuous variables undergo analysis with the Wilcoxon-Mann-Whitney test.

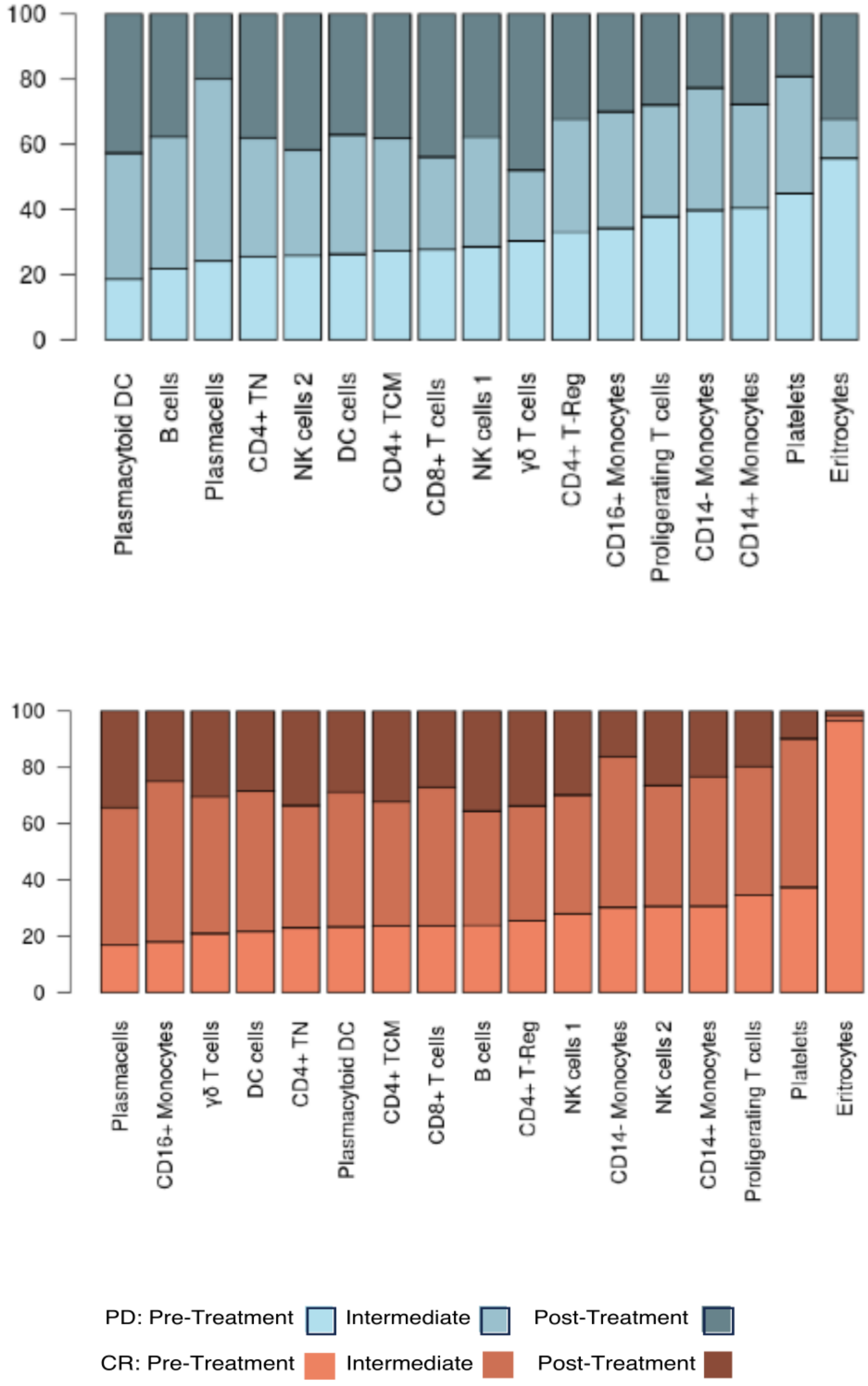

Supplementary Figures 1:

Barplot with proportion of each cell cluster in PD patients vs CR patients, in each stage of treatment.

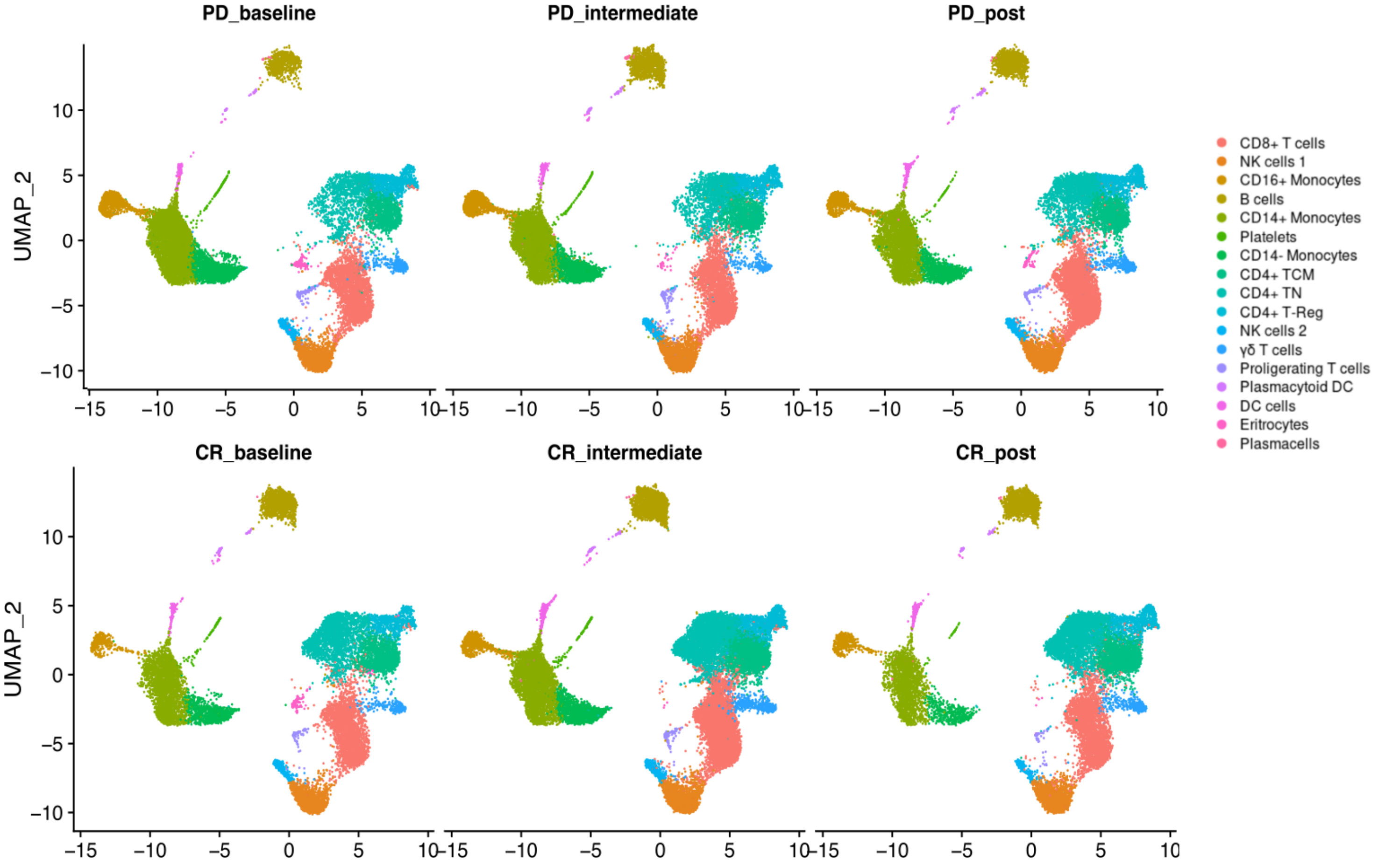

Supplementary Figures 2:

Two-dimensional similarity map (UMAP projection) of single-cell gene expression in different timepoints and different conditions. Cells are colored according to the PhenoGraph cluster. Subsets of cells are nominated according to their profile of gene markers.

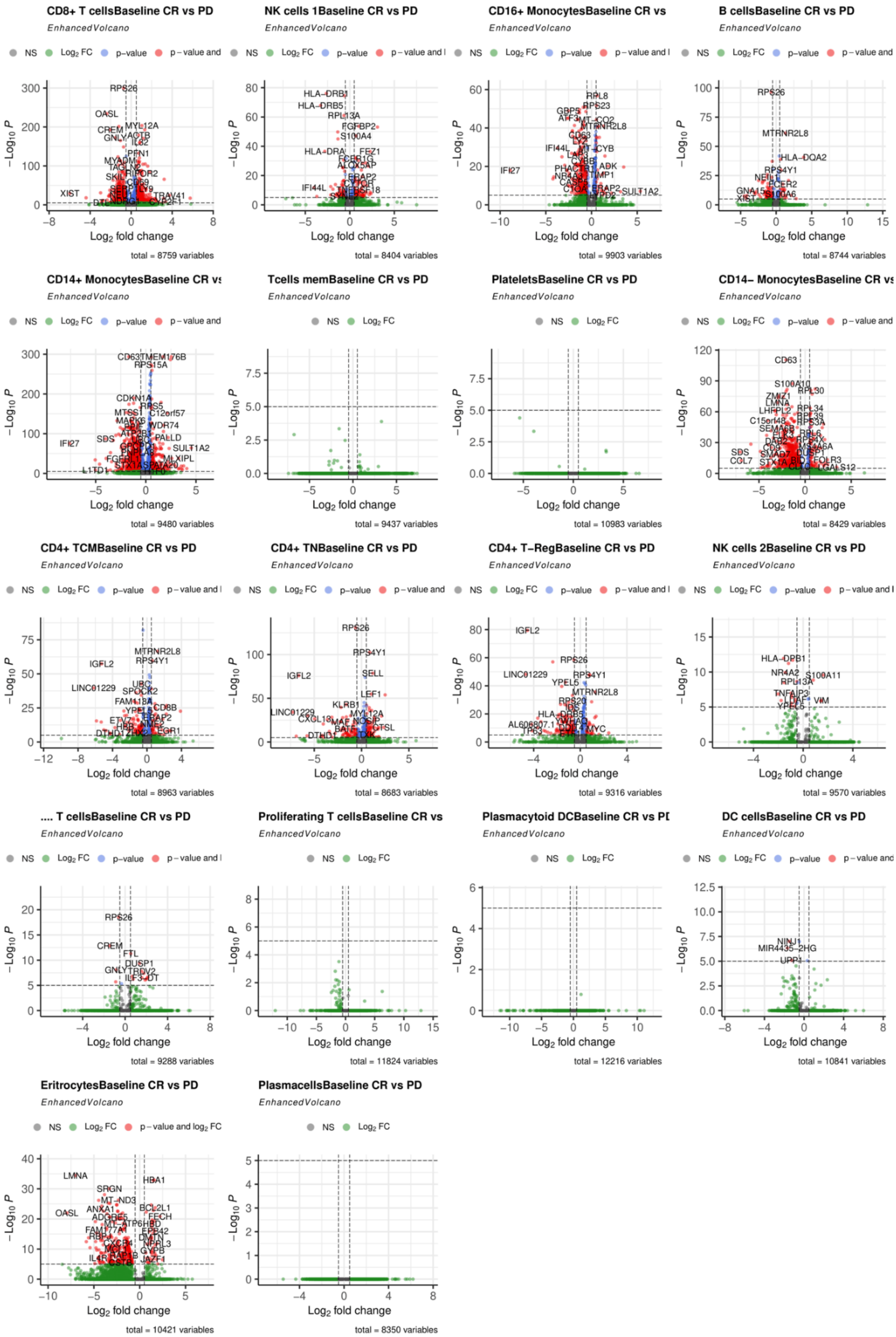
CD8+ T cellsBaseline CR vs PD
EnhancedVolcano
total = 8759 variables
NK cells 1Baseline CR vs PD
EnhancedVolcano
total = 8404 variables
CD16+ MonocytesBaseline CR vs
EnhancedVolcano
total = 9903 variables
B cellsBaseline CR vs PD
EnhancedVolcano
total = 8744 variables
CD14+ MonocytesBaseline CR vs
EnhancedVolcano
total = 9480 variables
Tcells memBaseline CR vs PD
EnhancedVolcano
total = 9437 variables
PlateletsBaseline CR vs PD
EnhancedVolcano
total = 10983 variables
CD14- MonocytesBaseline CR vs
EnhancedVolcano
total = 8429 variables
CD4+ TCMBaseline CR vs PD
EnhancedVolcano
total = 8963 variables
CD4+ TNBaseline CR vs PD
EnhancedVolcano
total = 8683 variables
CD4+ T-RegBaseline CR vs PD
EnhancedVolcano
total = 9316 variables
NK cells 2Baseline CR vs PD
EnhancedVolcano
total = 9570 variables
.... T cellsBaseline CR vs PD
EnhancedVolcano
total = 9288 variables
Proliferating T cellsBaseline CR vs
EnhancedVolcano
total = 11824 variables
Plasmacytoid DCBaseline CR vs PD
EnhancedVolcano
total = 12216 variables
DC cellsBaseline CR vs PD
EnhancedVolcano
total = 10841 variables
EritrocytesBaseline CR vs PD
EnhancedVolcano
total = 10421 variables
PlasmacellsBaseline CR vs PD
EnhancedVolcano
total = 8350 variables

Supplementary Figures 3a: Differential expression analysis in CD8+ T cells of CR patients at pre-treatment

Volcano plot showing differentially expressed genes between each cluster cells of responsive patients (CR) vs non-responsive patients (PD). Significant genes are labeled in red (P value <0.05 and absolute log2 fold change ≥0.5).

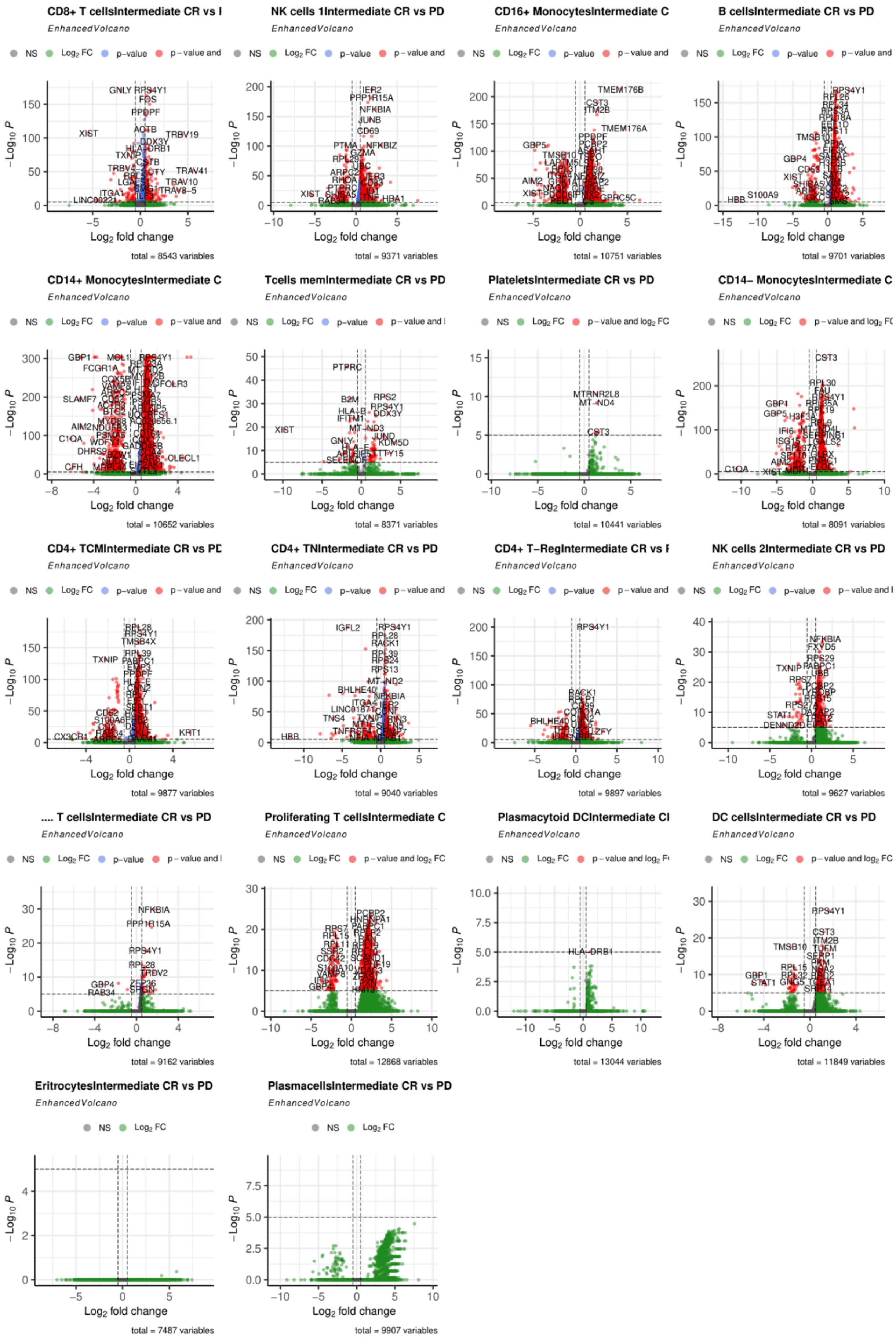
CD8+ T cellsIntermediate CR vs I
EnhancedVolcano
total = 8543 variables
NK cells 1Intermediate CR vs PD
EnhancedVolcano
total = 9371 variables
CD16+ MonocytesIntermediate C
EnhancedVolcano
total = 10751 variables
B cellsIntermediate CR vs PD
EnhancedVolcano
total = 9701 variables
CD14+ MonocytesIntermediate C
EnhancedVolcano
total = 10652 variables
Tcells memIntermediate CR vs PD
EnhancedVolcano
total = 8371 variables
PlateletsIntermediate CR vs PD
EnhancedVolcano
total = 10441 variables
CD14- MonocytesIntermediate C
EnhancedVolcano
total = 8091 variables
CD4+ TCMIntermediate CR vs PD
EnhancedVolcano
total = 9877 variables
CD4+ TNIntermediate CR vs PD
EnhancedVolcano
total = 9040 variables
CD4+ T-RegIntermediate CR vs I
EnhancedVolcano
total = 9897 variables
NK cells 2Intermediate CR vs PD
EnhancedVolcano
total = 9627 variables
.... T cellsIntermediate CR vs PD
EnhancedVolcano
total = 9162 variables
Proliferating T cellsIntermediate C
EnhancedVolcano
total = 12868 variables
Plasmacytoid DCIntermediate CI
EnhancedVolcano
total = 13044 variables
DC cellsIntermediate CR vs PD
EnhancedVolcano
total = 11849 variables
EritrocytesIntermediate CR vs PD
EnhancedVolcano
total = 7487 variables
PlasmacellsIntermediate CR vs PD
EnhancedVolcano
total = 9907 variables
NS
Log2 FC
p-value
p-value and log2 FC
Log2 fold change
-Log10 P

Supplementary Figures 3b: Differential expression analysis in CD8+ T cells of CR patients at intermediate stage

Volcano plot showing differentially expressed genes between each cluster cells of responsive patients (CR) vs non-responsive patients (PD). Significant genes are labeled in red (P value <0.05 and absolute log2 fold change ≥0.5)

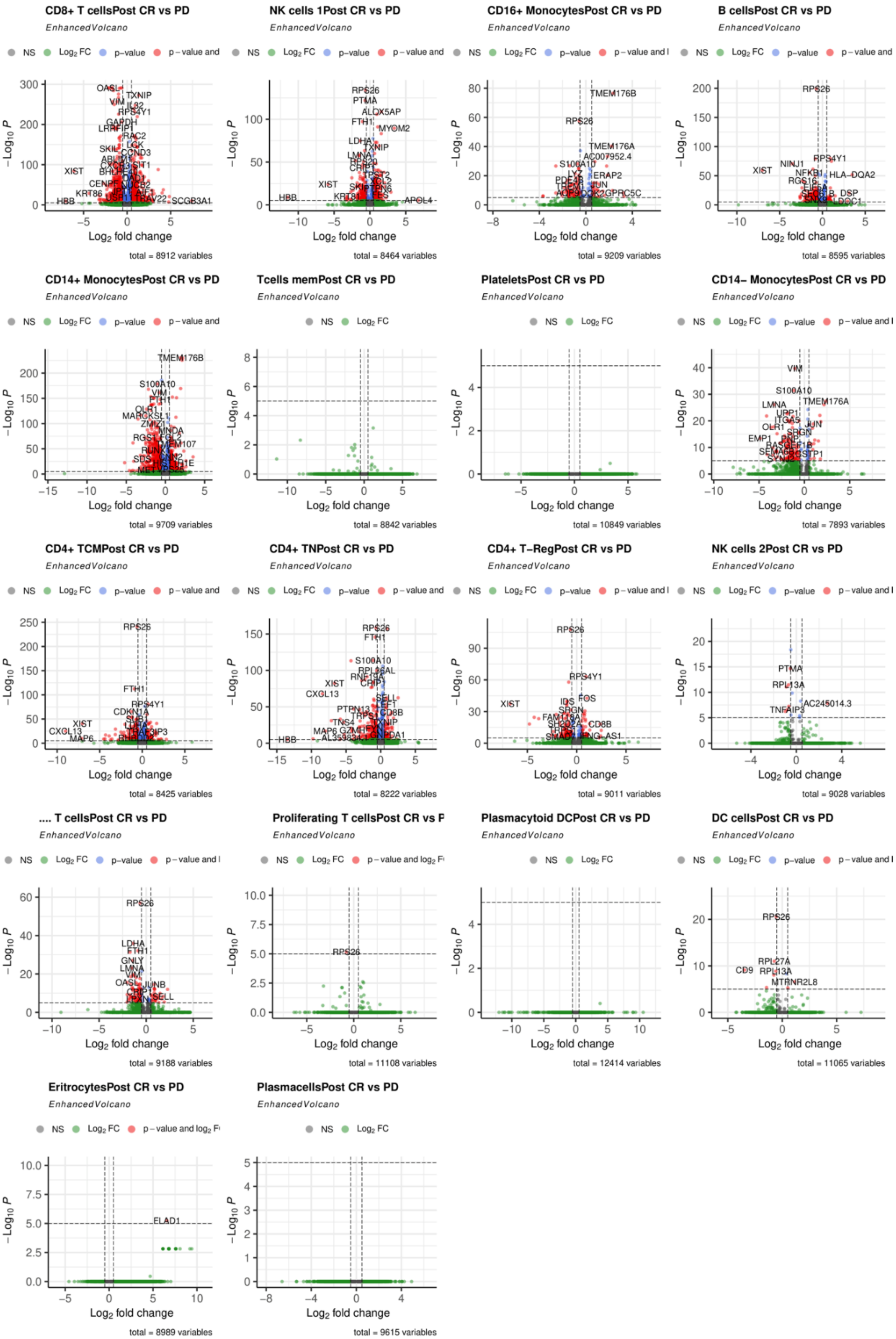

CD8+ T cellsPost CR vs PD
EnhancedVolcano
NS
Log2 FC
p−value
p − value and
OASL
TXNIP
VIM
RPS4Y1
GAPDH
LCK
CCND3
XIST
KRT86
HBB
SCGB3A1
Log2 fold change
total = 8912 variables
NK cells 1Post CR vs PD
EnhancedVolcano
RPS26
PTMA
ALOX5AP
FTH1
MYOM2
LDHA
TXNIP
LMNA
XIST
HBB
APOL4
Log2 fold change
total = 8464 variables
CD16+ MonocytesPost CR vs PD
EnhancedVolcano
TMEM176B
RPS26
TMEM176A
AC007952.4
S100A10
ERAP2
JUN
GPRC5C
Log2 fold change
total = 9209 variables
B cellsPost CR vs PD
EnhancedVolcano
RPS26
RPS4Y1
NINJ1
XIST
HLA−DQA2
DSP
Log2 fold change
total = 8595 variables
CD14+ MonocytesPost CR vs PD
EnhancedVolcano
TMEM176B
S100A10
VIM
FTH1
Log2 fold change
total = 9709 variables
Tcells memPost CR vs PD
EnhancedVolcano
Log2 fold change
total = 8842 variables
PlateletsPost CR vs PD
EnhancedVolcano
Log2 fold change
total = 10849 variables
CD14− MonocytesPost CR vs PD
EnhancedVolcano
VIM
S100A10
TMEM176A
LMNA
JUN
EMP1
Log2 fold change
total = 7893 variables
CD4+ TCMPost CR vs PD
EnhancedVolcano
RPS26
FTH1
RPS4Y1
CDKN1A
XIST
CXCL13
MAP6
Log2 fold change
total = 8425 variables
CD4+ TNPost CR vs PD
EnhancedVolcano
RPS26
FTH1
S100A10
XIST
CXCL13
TNS4
HBB
Log2 fold change
total = 8222 variables
CD4+ T−RegPost CR vs PD
EnhancedVolcano
RPS26
RPS4Y1
XIST
FOS
CD8B
Log2 fold change
total = 9011 variables
NK cells 2Post CR vs PD
EnhancedVolcano
PTMA
RPL13A
AC245014.3
TNFAIP3
Log2 fold change
total = 9028 variables
.... T cellsPost CR vs PD
EnhancedVolcano
RPS26
LDHA
FTH1
GNLY
LMNA
VIM
OASL
JUNB
SELL
Log2 fold change
total = 9188 variables
Proliferating T cellsPost CR vs P
EnhancedVolcano
RPS26
Log2 fold change
total = 11108 variables
Plasmacytoid DCPost CR vs PD
EnhancedVolcano
Log2 fold change
total = 12414 variables
DC cellsPost CR vs PD
EnhancedVolcano
RPS26
RPL27A
CD9
RPL13A
MTRNR2L8
Log2 fold change
total = 11065 variables
EritrocytesPost CR vs PD
EnhancedVolcano
ELAD1
Log2 fold change
total = 8989 variables
PlasmacellsPost CR vs PD
EnhancedVolcano
Log2 fold change
total = 9615 variables

Supplementary Figures 3c: Differential expression analysis in CD8+ T cells of CR patients at post-treatment

Volcano plot showing differentially expressed genes between each cluster cells of responsive patients (CR) vs non-responsive patients (PD). Significant genes are labeled in red (P value <0.05 and absolute log2 fold change ≥0.5).

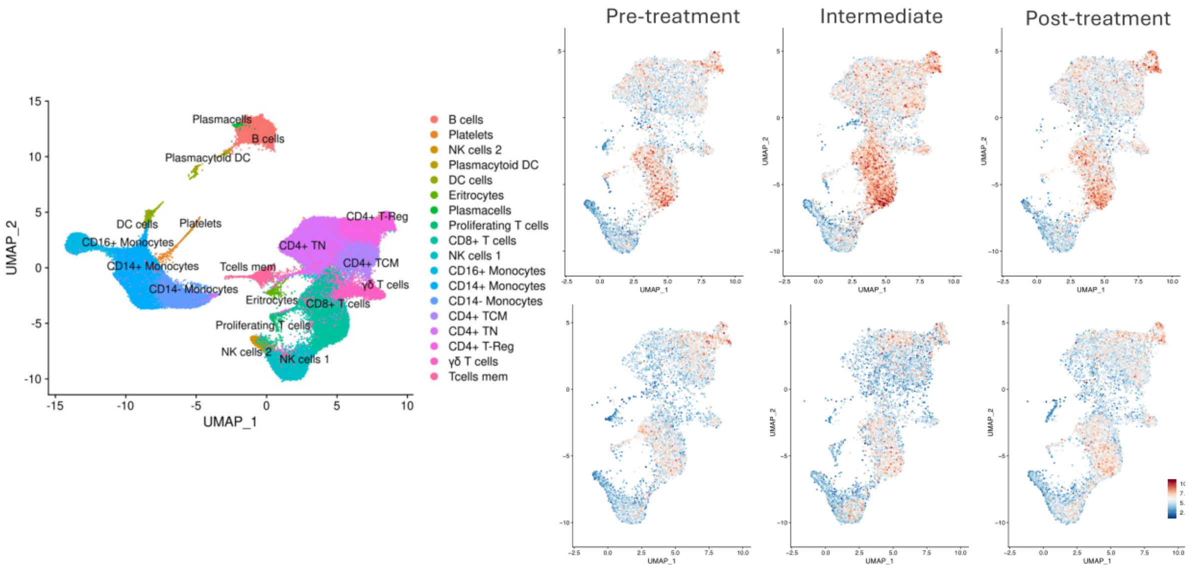

Supplementary Figure 4:

d) UMAP and signature related to TCR activation (GOCC: Alpha-beta TCR-Complex-GSEA dataset) splitted by condition..

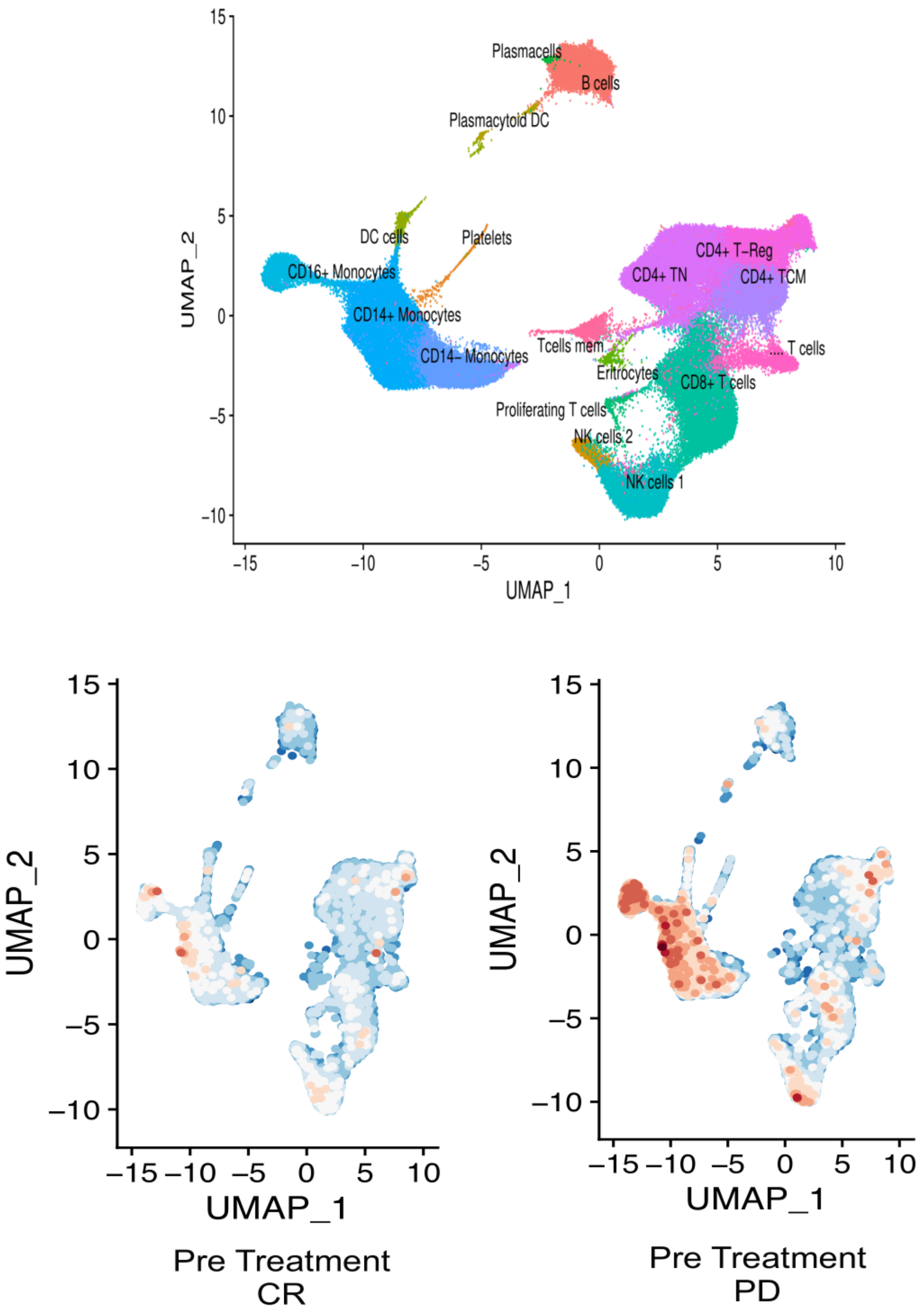

Supplementary Figure 5:

a) Combined two-dimensional similarity map (UMAP projection) of single-cell gene expression for all samples in the study cohort. **b)** UMAP projections with cells colored by module score analysis of pathway linked to the response to type I interferon at pre-treatment stage (GO:0034340), comparing CR baseline stage and PD Baseline stage.

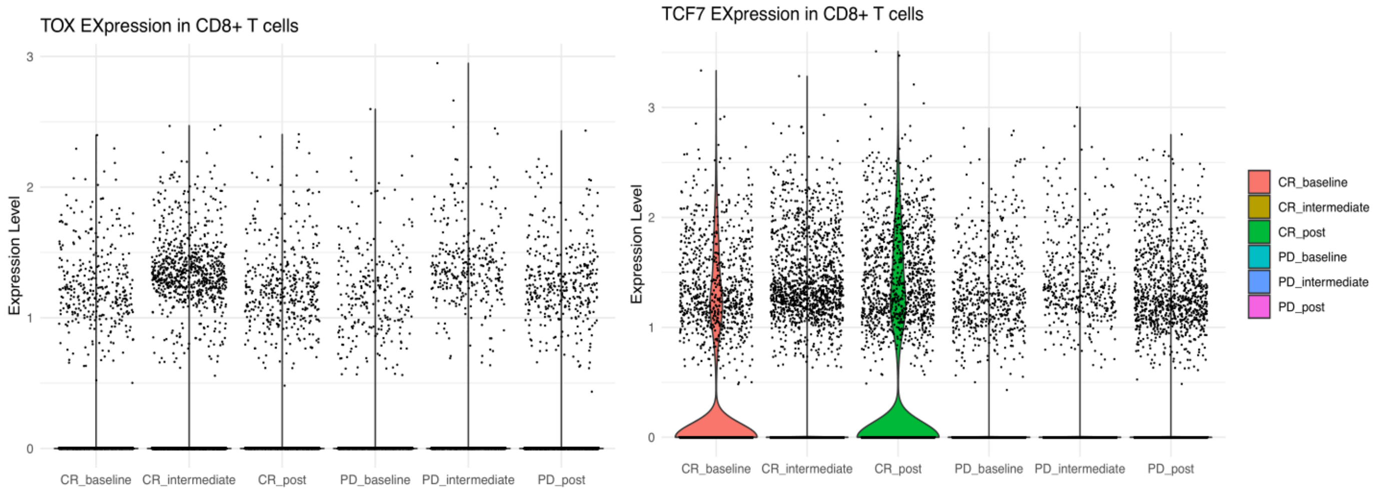

Supplementary Figure 6:

a) ViolinPlot showing exhausted markers expression (TOX , TCF7) in all condition.

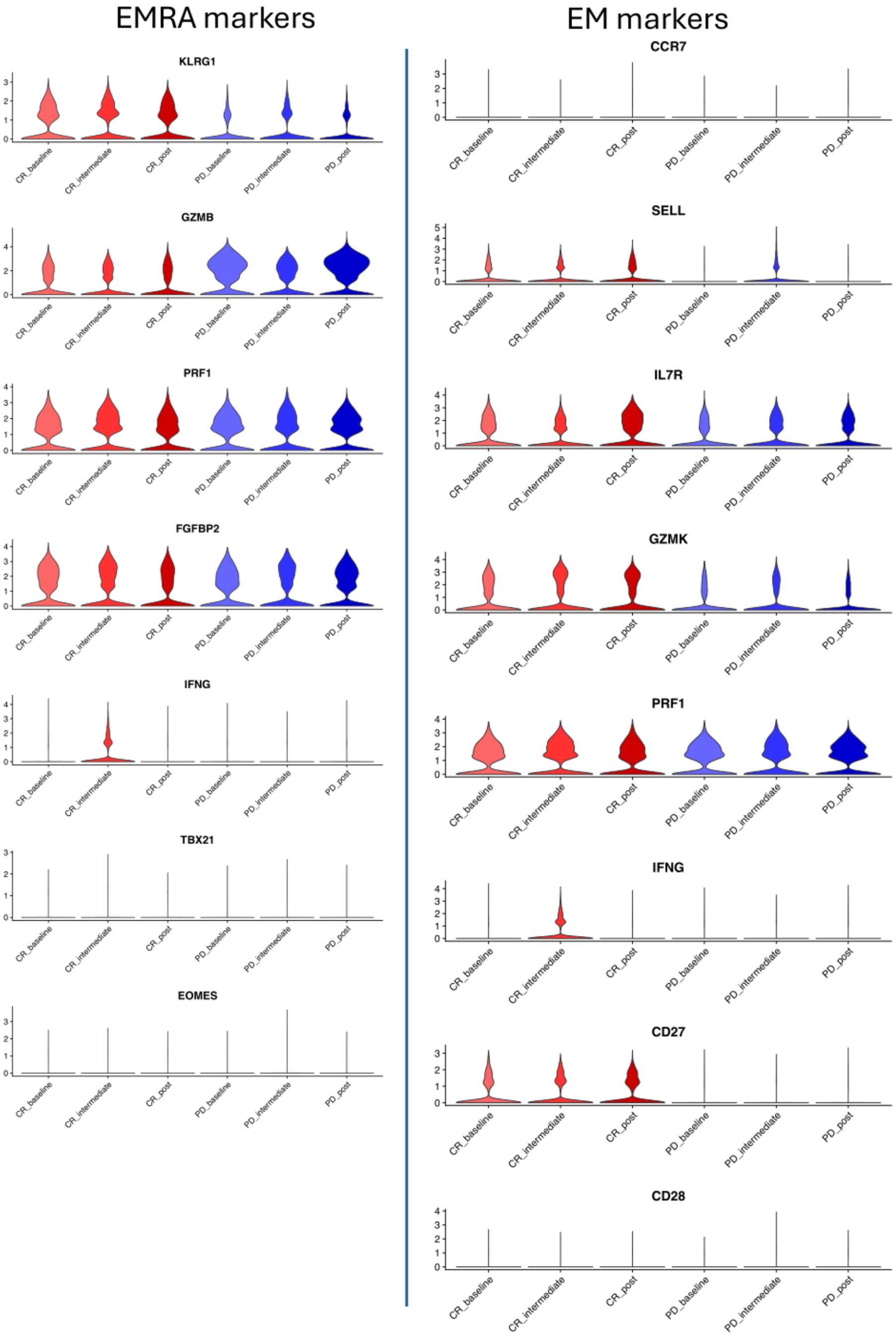

Supplementary Figure 7:

a) ViolinPlot showing markers of EMRA phenotype in CD8 T cells and EM phenotype in CD8 T cells splitted by condition

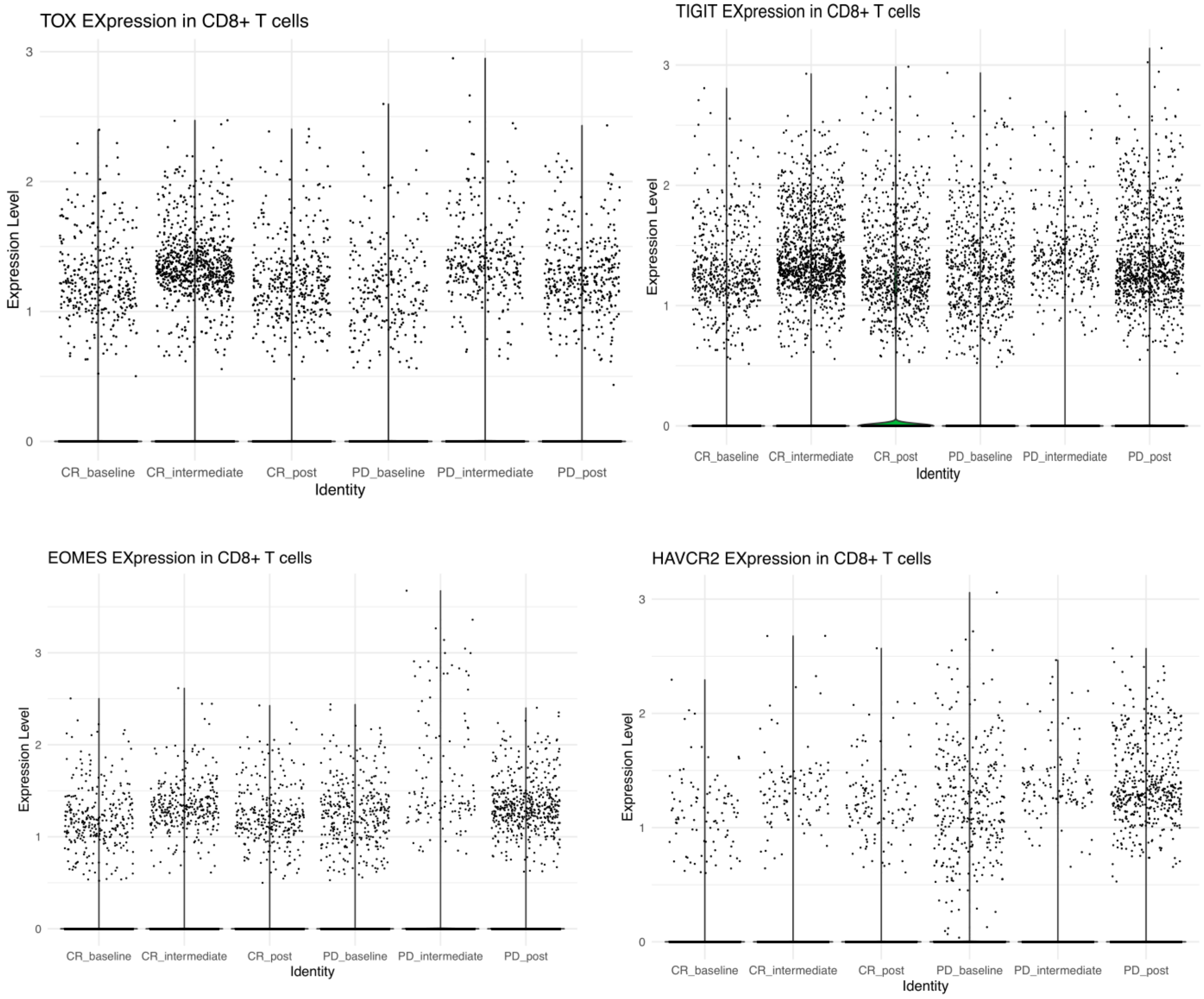

Supplementary Figure 8 :

a) ViolinPlot showing TOX, TIGIT ,EOMES and HAVCR2 expression in CD8 T cells splitted by condition.
